# Supplementary material for: The landscape of alternative polyadenylation in single cells of the developing mouse embryo
Source: Nat Commun. 2021 Aug 24;12:5101. doi: 10.1038/s41467-021-25388-8 (PMC8385098; doi:10.1038/s41467-021-25388-8)
Supplement: Supplementary file 1 — Supplementary Information [file 41467_2021_25388_MOESM1_ESM.pdf]

SUPPLEMENTARY TABLE 1

For Figure 2c

Cell type	P-value
Stromal cells	1.7e-33
Inhibitory neurons	3.0e-98
Excitatory neurons	1.7e-193
Postmitotic premature neurons	5.5e-137
Inhibitory interneurons	2.6e-37
Melanocytes	2.4e-04
Lens	1.0e-03
Inhibitory neuron progenitors	6.0e-77
Premature oligodendrocyte	7.7e-110
Chondrocytes and osteoblasts	<5.7e-308
Cholinergic neurons	1.0
Notochord cells	2.2e-40
Neural tube	6.1e-27
Neutrophils	0.25
Granule neurons	3.1e-43
Oligodendrocyte progenitors	1.7e-170
Sensory neurons	2.2e-121
Radial glia	1.4e-237
Limb mesenchyme	2.5e-81
Connective tissue progenitors	2.4e-144
Schwann cell precursor	1.2e-55
Cardiac muscle lineages	4.2e-34
Isthmic organizer cells	7.5e-121
Neural progenitor cells	1.6e-109
Ependymal cell	1.0e-33
Early mesenchyme	7.2e-54
Jaw and tooth progenitors	5.7e-308
Megakaryocytes	3.1e-15
Intermediate mesoderm	8.3e-259
Epithelial cells	6.4e-251
Myocytes	8.4e-72
Endothelial cells	8.6e-117
Primitive erythroid lineage	4.0e-25
Hepatocytes	9.6e-15
White blood cells	1.8e-33
Chondrocyte progenitors	6.4e-155
Osteoblasts	1.2e-115
Definitive erythroid lineage	6.0e-127

For Figure 3a

Trajectory	P-value
Neural tube and notochord	<5.7e-308
Neural crest (PNS neuron)	1.3e-4
Lens	7.7e-4
Neural crest (PNS glia)	1.5e-37
Neural crest melanocyte	2.2e-153
Mesenchymal	<5.7e-308
Epithelial	4.5e-192
Hematopoiesis	<5.7e-308
Endothelial	5.6e-95
Hepatocyte	2.5e-15

SUPPLEMENTARY TABLE 1 (continued)

For Figure 6c

Gene	P-value
Celf1	<2.2e-16
Celf2	<2.2e-16
Celf3	<2.2e-16
Celf4	<2.2e-16
Celf5	<2.2e-16
Celf6	<2.2e-16

For Figure 6d

Gene	P-value
Celf1	1.7e-02
Celf2	1.1e-01
Celf3	2.1e-07
Celf4	2.5e-06
Celf5	9.4e-06
Celf6	2.1e-07
Elavl1	1.0
Elavl2	2.1e-07
Elavl3	2.1e-07
Elavl4	2.1e-07
Nova1	7.6e-03
Nova2	4.2e-07
Rbfox1	4.0e-05
Rbfox2	5.5e-05
Rbfox3	4.2e-07
Hnrnpf	7.5e-05
Ptbp1	2.1e-07

For Supp. Figure 7b

Gene	P-value
Elavl1	<2.2e-16
Elavl2	<2.2e-16
Elavl3	<2.2e-16
Elavl4	<2.2e-16

For Supp. Figure 7c

Gene	P-value
Rbfox1	<2.2e-16
Rbfox2	<2.2e-16
Rbfox3	<2.2e-16

For Supp. Figure 7d

Gene	P-value
Hnrnpf	<2.2e-16
Ptbp1	<2.2e-16

Supplementary Table 1. Tables of exact p-values corresponding to **Figures 2c**, **3a**, **6c-d**, and **Supplementary Figure 7b-d**.

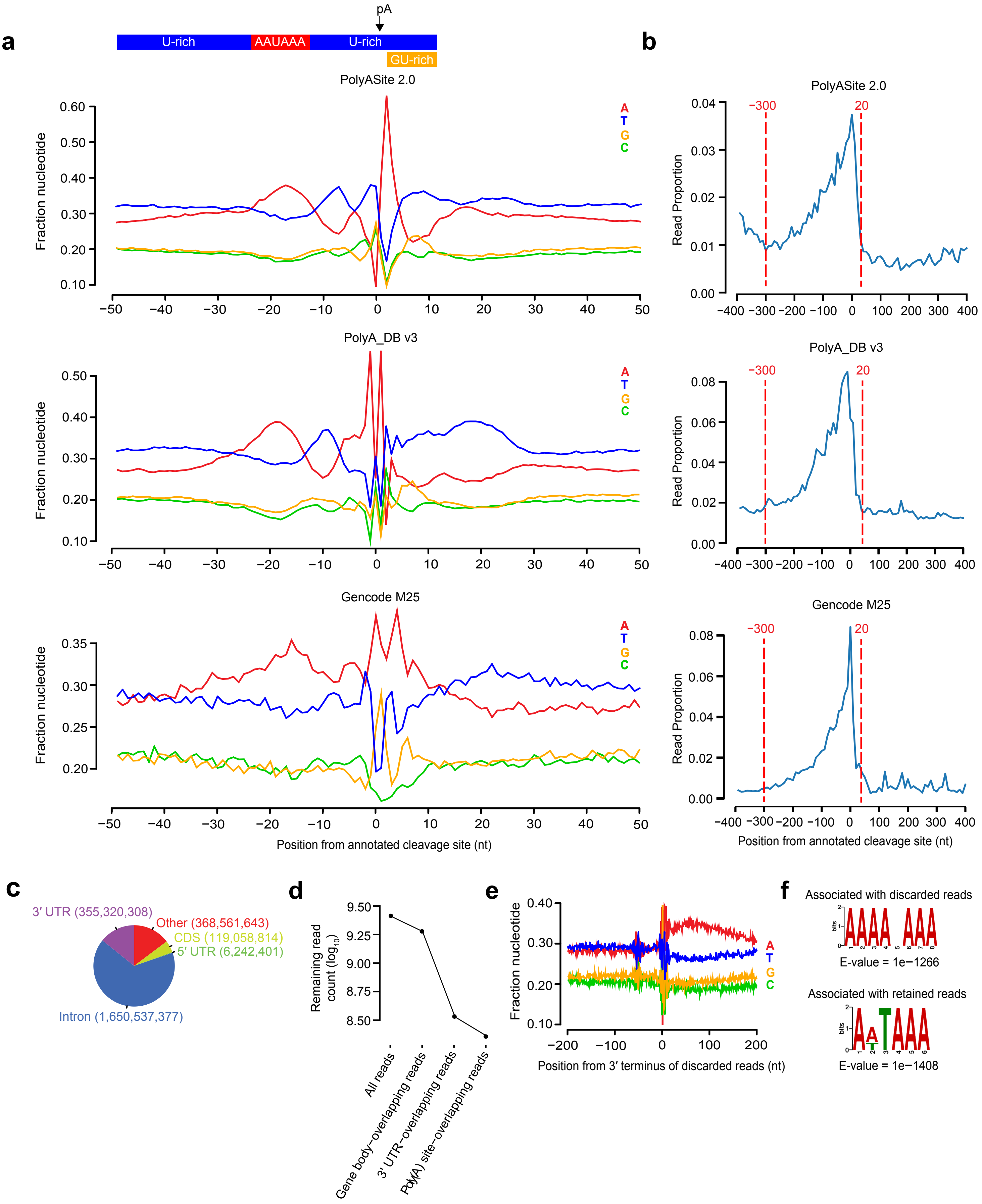

a
pA
U-rich
AAUAAA
U-rich
GU-rich
PolyASite 2.0
Fraction nucleotide
A
T
G
C
PolyA_DB v3
Gencode M25
Position from annotated cleavage site (nt)
b
PolyASite 2.0
−300
20
Read Proportion
PolyA_DB v3
Gencode M25
Position from annotated cleavage site (nt)
c
3′ UTR (355,320,308)
Other (368,561,643)
CDS (119,058,814)
5′ UTR (6,242,401)
Intron (1,650,537,377)
d
Remaining read count (log10)
All reads
Gene body–overlapping reads
3′ UTR–overlapping reads
Poly(A) site–overlapping reads
e
Fraction nucleotide
Position from 3′ terminus of discarded reads (nt)
f
Associated with discarded reads
E-value = 1e−1266
Associated with retained reads
E-value = 1e−1408

Supplementary Figure 1. Characterization of PAS annotation databases and properties of scRNA-seq data read filtering. (a) This panel is the same as that shown in **Fig. 1b**, except displays information for cleavage sites anchored on PASs unique to each of the three databases (*i.e.*, non-intersecting with a PAS from any other database). **(b)** This panel is the same as that shown in **Fig. 1c**, except displays information for cleavage sites anchored on PASs unique to each of the three databases (*i.e.*, non-intersecting with a PAS from any other database). **(c)** Pie chart showing the relative proportions of scRNA-seq reads that map to each functional region within the genome. **(d)** Plot of the decay in the numbers of reads remaining after each sequential filtering step that was required to isolate the subset of PAS-mapping reads. **(e)** This panel is the same as that shown in (a), except it plots the nucleotide composition in the ±200nt region relative to the 3′ terminus of 20,000 randomly sampled reads which were discarded by our filtering procedure. **(f)** Top-ranked enriched motif and corresponding E-value associated with the discarded reads in panel (e), as discovered by DREME v5.0.5[76] (top panel, parameters "-m8 -norc"). The discovered homopolymeric A-stretch was identified in the ±200nt window for 4958/20000 (24.8%) of reads. No AAUAAA motif was detected among the other 7 top-ranked motifs, suggesting the discarded reads were not significantly associated with cryptic PASs or those outside of the scope of our unified PAS database. Also shown is the top-ranked motif associated with a random sampling of 20,000 reads which passed our filters (bottom panel). Such reads were enriched near the AAUAAA motif as expected.

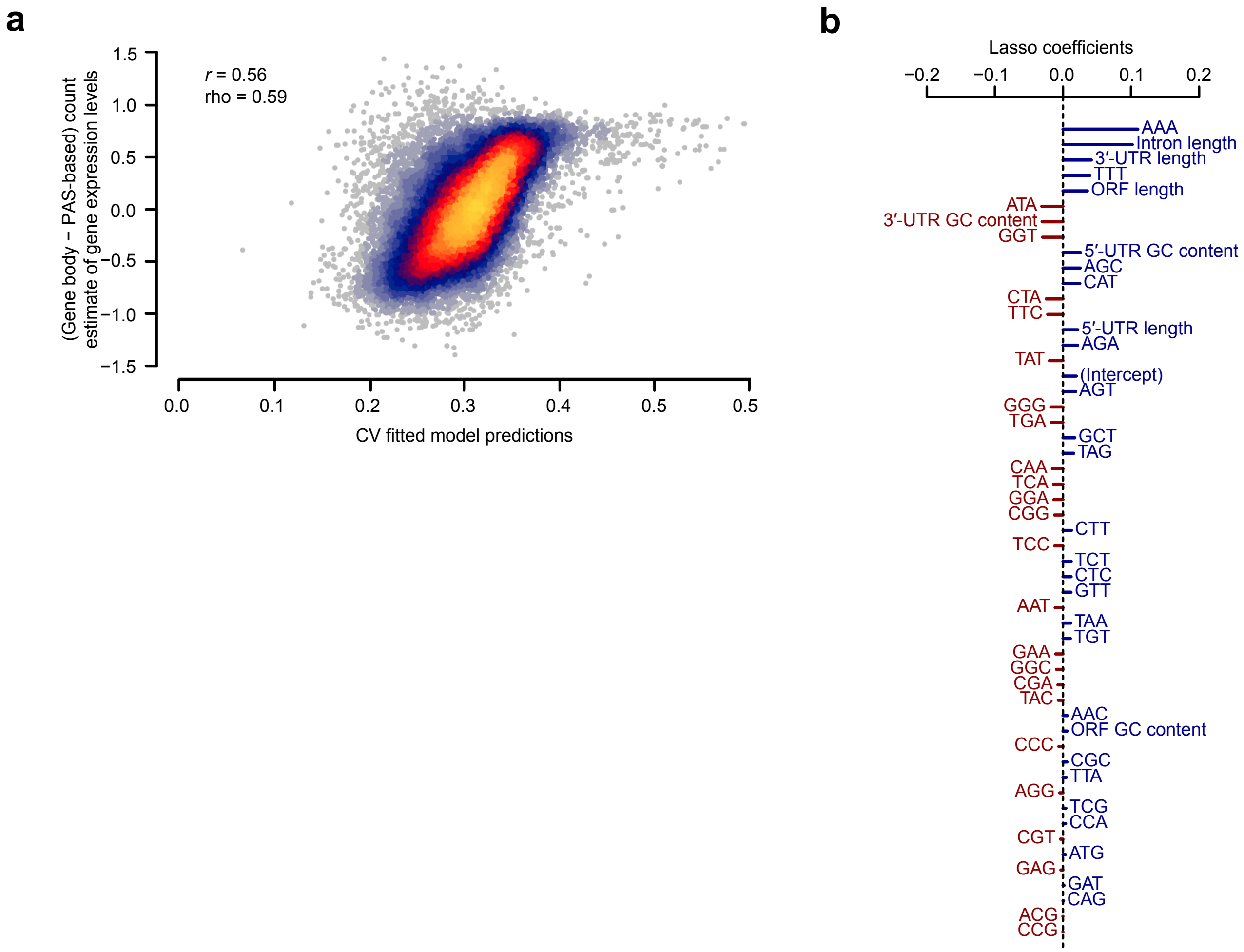

Supplementary Figure 2. Sequence-based features partially explain biases in the gene-body-based method of estimating relative gene expression levels. We developed a regression model to predict the difference between the gene body and PAS counting methods to estimate gene expression levels, as shown on the x-axes of the left and right panels, respectively, of **Fig. 1e**. The features considered in the model include the length of the 5′ UTR, ORF, introns, and 3′ UTR; the GC content of the 5′ UTR, ORF, and 3′ UTR; and the proportions of each of 64 possible 3-mers within the entire gene body. All features were z-score transformed to enable comparisons between regression coefficients. Following our previous work[77], we trained a lasso regression model using these features. The strength of the regularization was controlled by a single λ parameter, which was optimized using 10-fold cross-validation for each training set using the *cv.glmnet* function of the *glmnet* library in R. **(a)** Scatter plot displaying the relationship between the 10-fold cross-validated predictions derived from the lasso regression model and the observed difference between gene body and PAS-based counting methods of estimating gene expression level. Also indicated are the Pearson (r) and Spearman (rho) correlation values. **(b)** The ranked coefficients derived from a lasso regression model trained on the full dataset. Positive coefficients (blue) are associated with inflated gene body read counts, while negative coefficients (red) are associated with underrepresented gene body read counts.

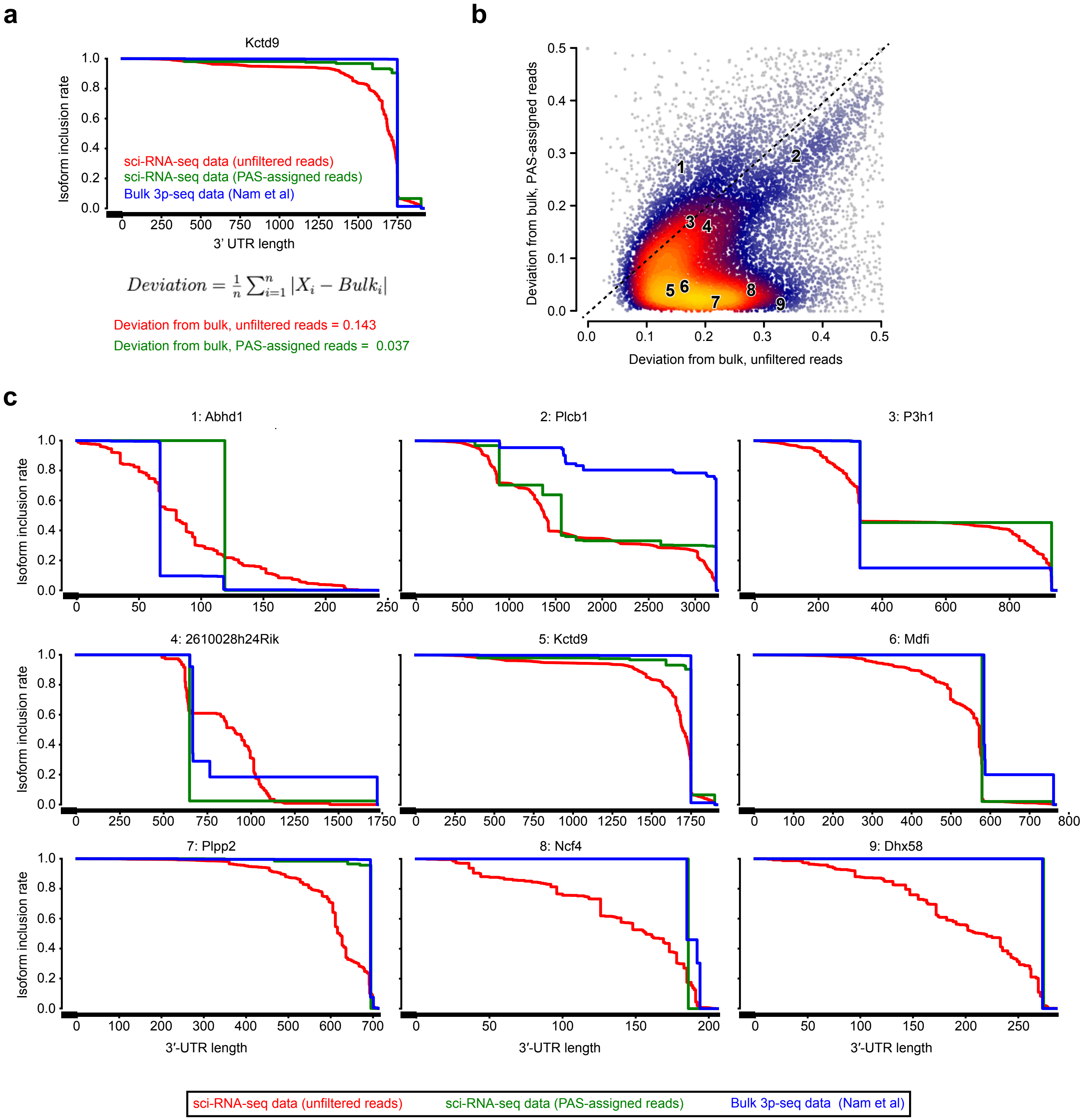
a
Kctd9
Isoform inclusion rate
sci-RNA-seq data (unfiltered reads)
sci-RNA-seq data (PAS-assigned reads)
Bulk 3p-seq data (Nam et al)
3' UTR length
Deviation = 1/n Σ_{i=1}^{n} |X_i − Bulk_i|
Deviation from bulk, unfiltered reads = 0.143
Deviation from bulk, PAS-assigned reads = 0.037
b
Deviation from bulk, PAS-assigned reads
Deviation from bulk, unfiltered reads
c
1: Abhd1
2: Plcb1
3: P3h1
4: 2610028h24Rik
5: Kctd9
6: Mdfi
7: Plpp2
8: Ncf4
9: Dhx58
Isoform inclusion rate
3′-UTR length
sci-RNA-seq data (unfiltered reads)
sci-RNA-seq data (PAS-assigned reads)
Bulk 3p-seq data (Nam et al)

Supplementary Figure 3. Improvement in the quantification of 3′-UTR isoform abundances after read-to-PAS assignment. (a) Calculation of the Mean Absolute Deviation (MAD) metric on an example gene to quantify the divergence between IIR profiles derived from either raw scRNA-seq data or post-processed data after read-to-PAS assignment, relative to the profile for bulk 3P-seq data[8] as a gold standard. Larger numbers indicate poorer agreement. **(b)** Scatter plot of MAD values for either raw scRNA-seq data (x-axis) or post-processed data after read-to-PAS assignment (y-axis) (n = 16,334 protein-coding genes). Regions are colored according to the density of data from light blue (low density) to yellow (high density). 78% of genes exist below the diagonal dotted line, indicating an improved similarity to bulk measurements after post-processing. Nine genes from different representative regions of the plot are indicated. **(c)** IIR plots for the nine representative genes numbered in panel (b). The majority of genes show strongly improved agreement to bulk 3P-seq data (genes 5-9).

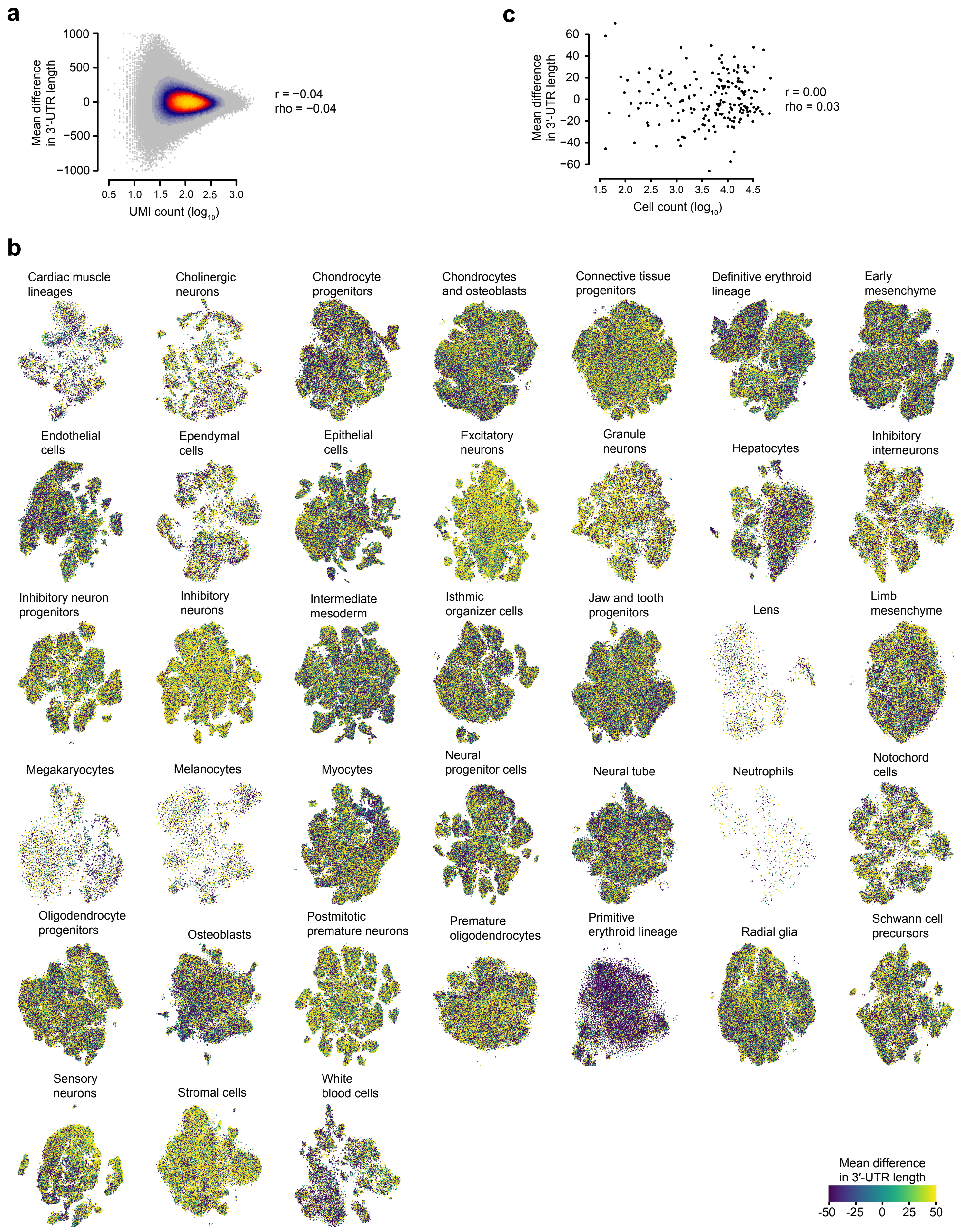
a
Mean difference in 3′-UTR length
UMI count (log10)
r = −0.04
rho = −0.04
c
Mean difference in 3′-UTR length
Cell count (log10)
r = 0.00
rho = 0.03
b
Cardiac muscle lineages
Cholinergic neurons
Chondrocyte progenitors
Chondrocytes and osteoblasts
Connective tissue progenitors
Definitive erythroid lineage
Early mesenchyme
Endothelial cells
Ependymal cells
Epithelial cells
Excitatory neurons
Granule neurons
Hepatocytes
Inhibitory interneurons
Inhibitory neuron progenitors
Inhibitory neurons
Intermediate mesoderm
Isthmic organizer cells
Jaw and tooth progenitors
Lens
Limb mesenchyme
Megakaryocytes
Melanocytes
Myocytes
Neural progenitor cells
Neural tube
Neutrophils
Notochord cells
Oligodendrocyte progenitors
Osteoblasts
Postmitotic premature neurons
Premature oligodendrocytes
Primitive erythroid lineage
Radial glia
Schwann cell precursors
Sensory neurons
Stromal cells
White blood cells
Mean difference in 3′-UTR length
-50
-25
0
25
50

Supplementary Figure 4. Analysis of potential confounding biases and differential 3′-UTR lengthening among cellular subtypes. (a) Scatter plot showing the relationship between the number of reads, computed as unique molecular indexes (UMIs), relative to the mean change in 3′-UTR length among cells as shown in **Fig. 2a**. Regions are colored according to the density of data from grey and blue (low density) to yellow (high density). Also listed are the Pearson (*r*) and Spearman (rho) correlations. **(b)** t-SNE embeddings were generated to identify cellular subtypes for cell types derived from each of 38 t-SNE clusters[31]. Each cell was colored according to the mean difference in 3′-UTR lengths across all genes. Local subcluster heterogeneity can be observed (*e.g.*, cellular subtypes in hepatocytes and osteoblasts), along with global differences between clusters (*e.g.*, the primitive erythroid lineage relative to neuronal cells and stromal cells). **(c)** Scatter plot showing the relationship between the number of cells relative to the mean change in 3′-UTR length among developmental stage and cell type bins as shown in **Fig. 2c**. Also listed are the Pearson (*r*) and Spearman (rho) correlations.

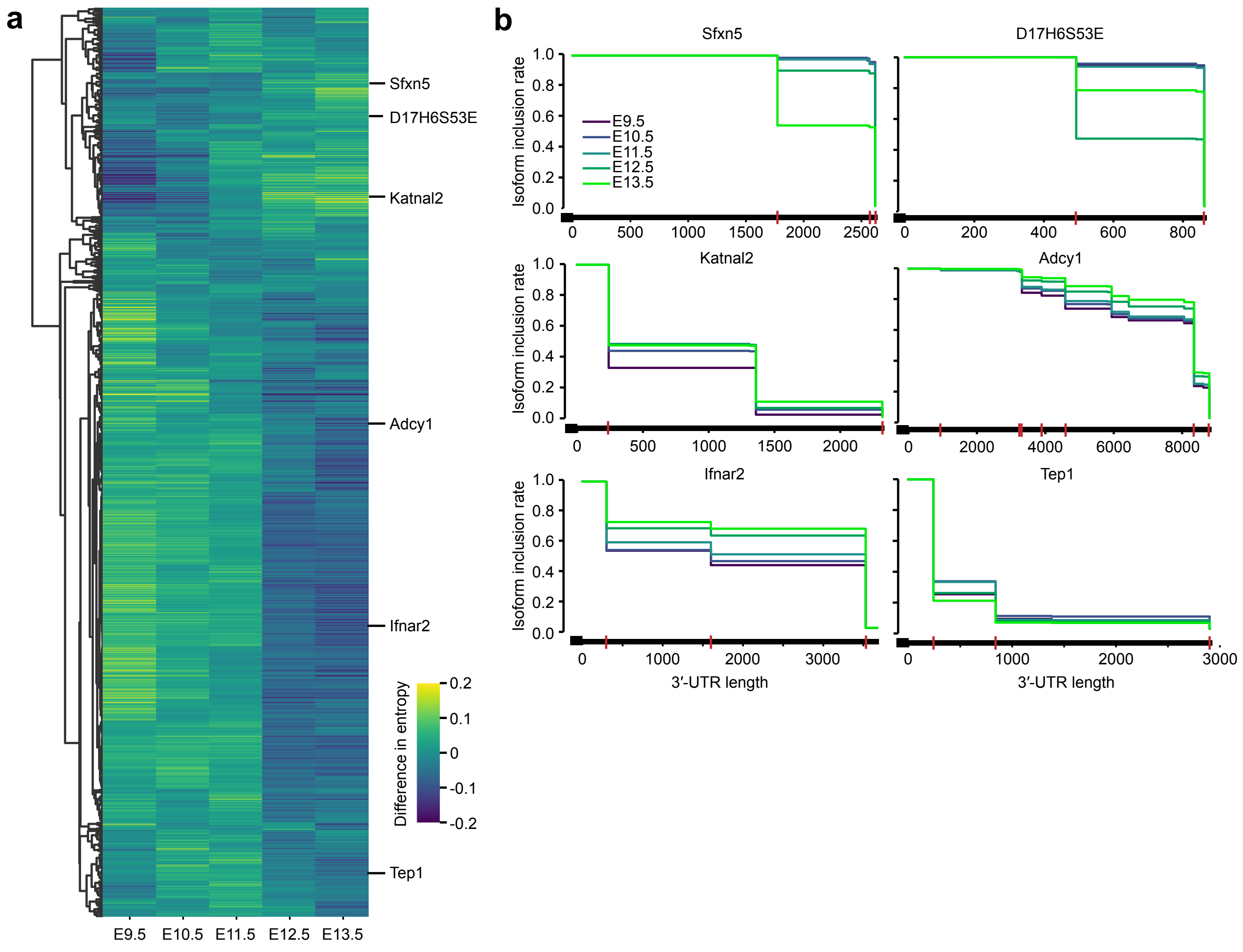

Supplementary Figure 5. Evaluation of differential PAS usage and diversity across developmental time. **(a)** Heatmap of differences in entropy for the statistically significant genes from **Fig. 4a**. Entropy for a given gene and developmental stage was calculated as $H(x) = (-\sum_{(x \in PAS)} P(x)\ log_2\ P(x))$ using relative read proportions assigned to each PAS for the set of all PASs associated with the gene. Heatmap is row-centered and clustered by Pearson correlation as a distance metric. Higher values of entropy represent a greater degree of randomness and uniformity in selection among multiple PASs; conversely, lower values represent greater fidelity in selection amongst fewer PASs. **(b)** IIR plots for six genes among representative clusters shown in panel (a), and colored by developmental stage. Vertical red lines along the 3′ UTR indicate PASs that are significantly different between stages by the χ^2 test ($p<0.05$).

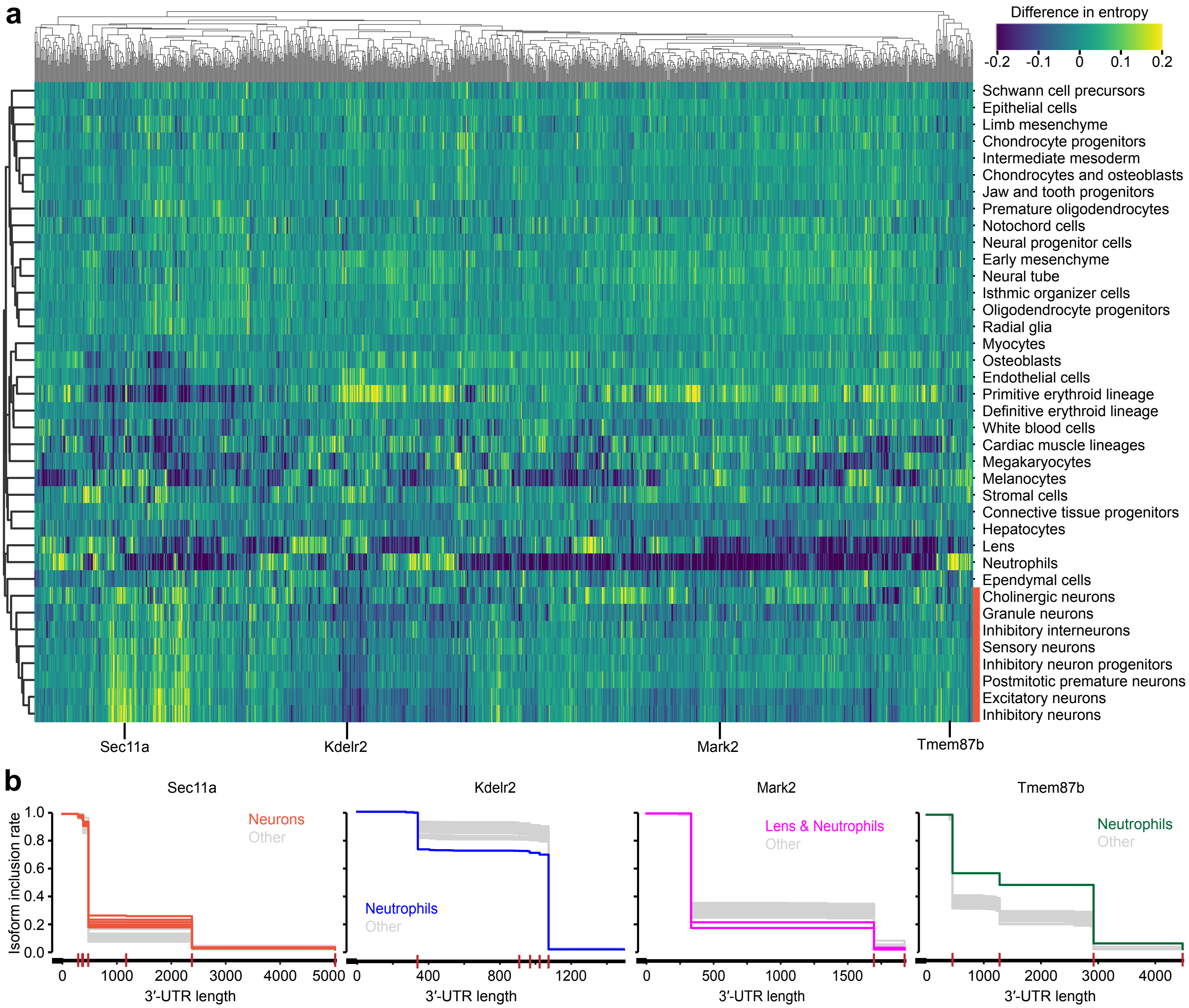

Supplementary Figure 6. Evaluation of differential PAS usage and diversity across cell types. (a) Heatmap of differences in entropy for the statistically significant genes from **Fig. 5a**. Entropy for a given gene and cell type (derived from each of 38 t-SNE clusters) was calculated as $H(x) = (-\sum_{(x \in \text{PAS})} P(x) \log_2 P(x))$ using relative read proportions assigned to each PAS for the set of all PASs associated with the gene. Heatmap is column-centered and clustered in both rows and columns by Pearson correlation as a distance metric. Higher values of entropy represent a greater degree of randomness and uniformity in selection among multiple PASs; conversely, lower values represent greater fidelity in selection amongst fewer PASs. **(b)** IIR plots for five genes among representative clusters shown in panel (a), and colored either by the indicated cell types or the cluster of neuronal cell types shown in panel (a). Grey lines allude to all other cell types. Vertical red lines along the 3′ UTR indicate PASs that are significantly different between cell types by the χ^2 test ($p<0.05$).

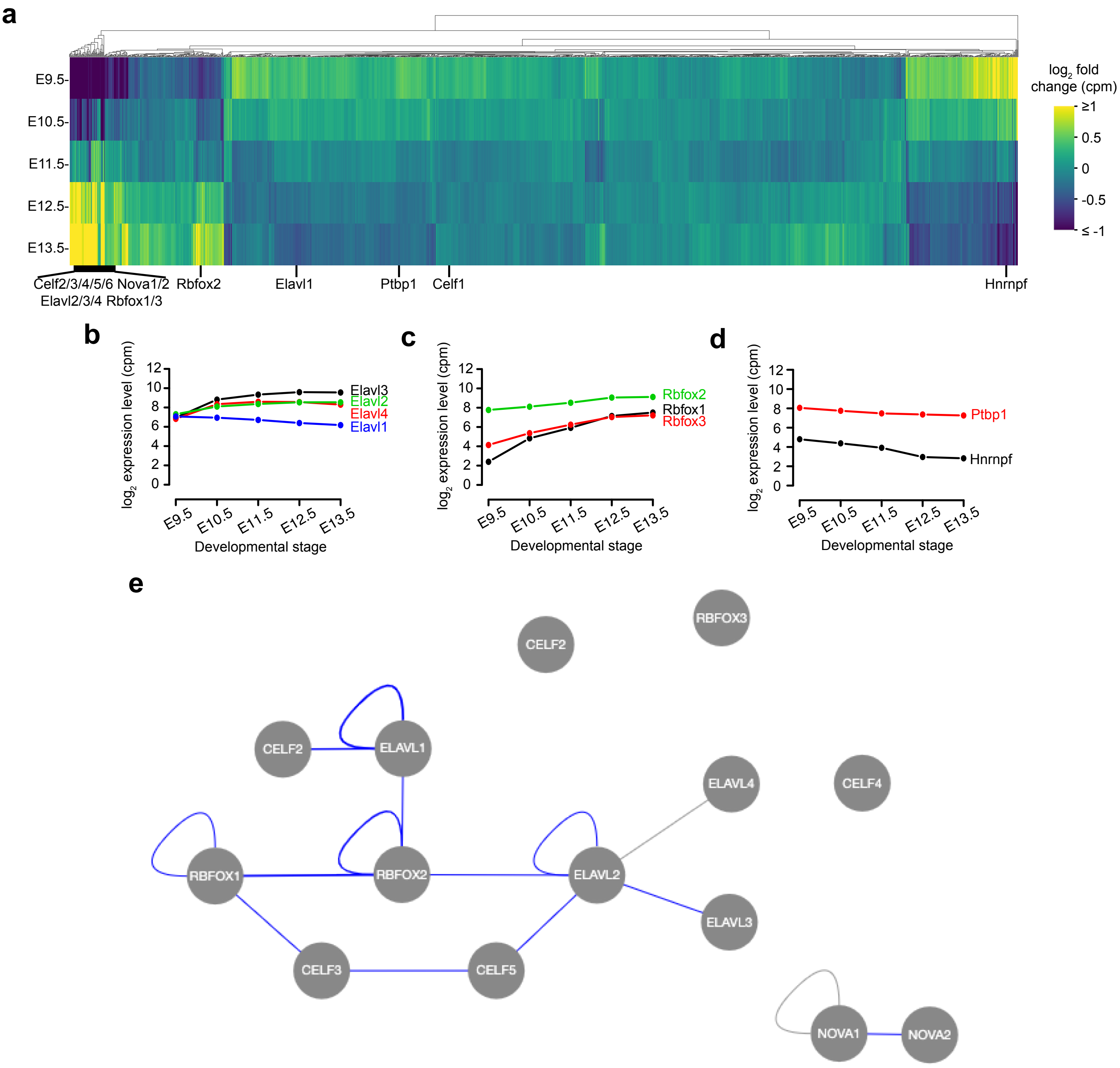

Supplementary Figure 7. **Evaluation of differential RNA-binding protein expression across developmental time. (a)** Heatmap of relative gene expression levels, quantified as $\log_2$(counts per million), for a set of 1,516 RBPs across the five embryonic stages. Heatmap is column-centered and clustered by Euclidean distance as a distance metric. **(b-d)** Expression levels of *Elavl1-4* (b), *Rbfox1-3* (c), and *Ptbp1/Hnrnpf* (d) across the five developmental stages. Expression is quantified in counts per million (cpm) and shown on a $\log_2$ scale. All genes displayed significant differential expression across stages, with a Bonferroni-corrected $p<0.001$ as assessed by the χ^2 test for homogeneity, using PAS-mapping read counts associated with the RBP of interest versus all other RBPs. Exact p-values are provided in **Supplementary Table 1**. **(e)** Network of protein-protein interactions supporting RBP interactions as observed in the APID database[46]. Blue lines indicate additional experimental support for the interaction.
