# Supplementary material for: The landscape of alternative polyadenylation in single cells of the developing mouse embryo
Source: Nat Commun. 2021 Aug 24;12:5101. doi: 10.1038/s41467-021-25388-8 (PMC8385098; doi:10.1038/s41467-021-25388-8)
Supplement: Supplementary file 11 — Description of additional supplementary files [file 41467_2021_25388_MOESM11_ESM.docx]

**Description of additional supplementary files**

**Supplementary Data 1.** GTF file of the gene models associated with the “Integrated 3′ UTR” set, as described in **Fig. 1d**, along with the source database of the 3′-UTR annotation. Genomic coordinates are provided with respect to the mm10 genome.

**Supplementary Data 2.** Table of FDR-corrected q-values for each transcript/gene tested for differential PAS usage across the five developmental stages. Genes that did not pass the threshold of having 100 reads at each age are listed as “Not Tested”.

**Supplementary Data 3.** Table of read counts associated with each PAS for each gene and developmental stage. Genomic coordinates are provided for every PAS with respect to the mm10 genome, along with the annotation source of the PAS.

**Supplementary Data 4.** Table of FDR-corrected q-values for each transcript/gene tested for differential PAS usage across the 38 cell types. Genes that did not pass the threshold of having 20 reads in each cell type are listed as “Not Tested”.

**Supplementary Data 5.** Table of read counts associated with each PAS for each gene and cell type. Genomic coordinates are provided for every PAS with respect to the mm10 genome, along with the annotation source of the PAS.

**Supplementary Data 6.** Table of expression levels of protein-coding genes, computed as counts per million, across the five developmental stages. Genes are annotated according to which correspond to known RNA binding proteins.

**Supplementary Data 7.** Table of expression levels of protein-coding genes, computed as counts per million, across the 38 cell types. Genes are annotated according to which correspond to known RNA binding proteins.

**Supplementary Data 8.** Table of log_2_(fold changes) of expression levels for RBPs tested in E13.5 vs E9.5 and in neuronal lineages vs other cell types, ranked by their degree of upregulation in neurons.
